# *Aggregatibacter actinomycetemcomitans* Leukotoxin Is Delivered to Host Cells in an LFA-1-Indepdendent Manner When Associated with Outer Membrane Vesicles

**DOI:** 10.3390/toxins10100414

**Published:** 2018-10-13

**Authors:** Justin B. Nice, Nataliya V. Balashova, Scott C. Kachlany, Evan Koufos, Eric Krueger, Edward T. Lally, Angela C. Brown

**Affiliations:** 1 Department of Chemical and Biomolecular Engineering, Lehigh University, Bethlehem, PA 18015, USA; jbn212@lehigh.edu (J.B.N.); evk213@lehigh.edu (E.K.); erk415@lehigh.edu (E.K.); 2 Department of Pathology, University of Pennsylvania School of Dental Medicine, Philadelphia, PA 19104, USA; natbal@upenn.edu (N.V.B.); lally@upenn.edu (E.T.L.); 3 Department of Oral Biology, Rutgers University School of Dental Medicine, Newark, NJ 07101, USA; kachlasc@sdm.rutgers.edu

**Keywords:** *Aggregatibacter actinomycetemcomitans*, outer membrane vesicles, leukotoxin, cholesterol, lymphocyte function-associated antigen-1 (LFA-1), RTX toxin

## Abstract

The Gram-negative bacterium, *Aggregatibacter actinomycetemcomitans,* has been associated with localized aggressive periodontitis (LAP). In particular, highly leukotoxic strains of *A. actinomycetemcomitans* have been more closely associated with this disease, suggesting that LtxA is a key virulence factor for *A. actinomycetemcomitans*. LtxA is secreted across both the inner and outer membranes via the Type I secretion system, but has also been found to be enriched within outer membrane vesicles (OMVs), derived from the bacterial outer membrane. We have characterized the association of LtxA with OMVs produced by the highly leukotoxic strain, JP2, and investigated the interaction of these OMVs with host cells to understand how LtxA is delivered to host cells in this OMV-associated form. Our results demonstrated that a significant fraction of the secreted LtxA exists in an OMV-associated form. Furthermore, we have discovered that in this OMV-associated form, the toxin is trafficked to host cells by a cholesterol- and receptor-independent mechanism in contrast to the mechanism by which free LtxA is delivered. Because OMV-associated toxin is trafficked to host cells in an entirely different manner than free toxin, this study highlights the importance of studying both free and OMV-associated forms of LtxA to understand *A. actinomycetemcomitans* virulence.

## 1. Introduction

*Aggregatibacter actinomycetemcomitans* is associated with localized aggressive periodontitis (LAP) [1], a disease characterized by rapid and extensive alveolar bone loss [2,3], as well as with systemic diseases including endocarditis [4]. The current standard treatment of LAP involves debridement in combination with systemic antibiotics, such as tetracycline. This treatment is ineffective for up to 25% of patients, [5,6,7] which has been attributed to increased antibiotic resistance in *A. actinomycetemcomitans* [8,9]. *A. actinomycetemcomitans* produces a number of virulence factors [10], including a leukotoxin (LtxA), which specifically kills host immune cells, providing a colonization advantage for the bacterium [11]. Defining the mechanisms by which LtxA is delivered to host cells could enable the identification of new therapeutic targets for LAP.

Like other members of the repeats-in-toxin (RTX) family of proteins, LtxA is secreted through a one-step Type I secretion system, in which the toxin is transported across the inner and outer membranes in a single step without a periplasmic intermediate [12,13]. In its secreted water-soluble form, LtxA has been demonstrated to bind to an integrin receptor, lymphocyte function-associated antigen-1 (LFA-1) [14,15,16], which is only expressed by human white blood cells, an interaction that provides the toxin with its cell type specificity, in addition to the cell death receptor, Fas [17]. We have demonstrated that LtxA also recognizes cholesterol on the host cell plasma membrane [18,19] via a cholesterol recognition amino acid consensus motif [19] and disrupts bilayer packing of the plasma membrane by inducing nonlamellar phase formation [20,21,22]. In THP-1 cells and erythroleukemia cells, LtxA is then internalized in a lysosome-mediated mechanism [23,24].

In addition to this secretion into the extracellular environment as a free-floating, water-soluble protein, LtxA, like other members of the RTX toxin family, is also released from the bacterial cell in association with outer membrane vesicles (OMVs) [25,26,27,28,29,30], which are formed from the outer membrane (OM) of Gram-negative bacteria [31,32,33]. In recent years, significant evidence has accumulated linking OMVs to virulence [28,34,35,36,37,38,39,40,41,42,43,44] through their ability to enrich and protect virulence factors and deliver them over longer distances than they would otherwise be able to travel [45]. In *A. actinomycetemcomitans*, reports have linked the extent of vesiculation to the expression of LtxA, with highly leukotoxic strains producing significantly more vesicles than minimally or nonleukotoxic strains [46,47]. Another intriguing discovery is the finding that the OMVs of LtxA-producing strains of *A. actinomycetemcomitans* are enriched in LtxA relative to the bacterial cell [27], suggesting a link between LtxA and OMV biogenesis. However, LtxA is not the only active molecule in *A. actinomycetemcomitans* OMVs. OMVs produced by strain D7SS belonging to serotype a have been shown to include active cytolethal distending toxin (Cdt) [48], as well as to deliver peptidoglycan to the cytosol to initiate NOD1-dependent NF-κB activation [49]. *A. actinomycetemcomitans* OMVs have also been reported to carry several molecules involved in bone resorption, including lipopolysaccharide (LPS) [50,51] and lipid A-associated proteins [52], as well as numerous virulence factors [53].

Our lab has used knowledge of the mechanism by which free LtxA interacts with host cells to engineer small peptides to block specific aspects of the toxin’s mechanism in order to inhibit LtxA-mediated cytotoxicity. In particular, we have successfully inhibited both the toxin’s recognition of cholesterol [54,55] and the CD11a subunit of LFA-1 [56] to inhibit LtxA-mediated cytotoxicity. However, the fact that *A. actinomycetemcomitans* OMVs can be delivered in an LtxA-independent manner suggests that in this membrane-associated form, LtxA may be delivered to the cell in a cholesterol and/or LFA-1-independent mechanism, a possibility with important implications in the design of agents to inhibit LtxA activity. In order to fully inhibit LtxA activity as an anti-virulence strategy we must, therefore, understand how LtxA is delivered to host cells when it is in this OMV-associated form. Therefore, our goal in this project was to characterize delivery of LtxA to host immune cells via OMVs to determine if this delivery system accounts for a significant fraction of LtxA delivery to host cells and to establish whether the receptors of purified LtxA, LFA-1 and cholesterol, play a role in OMV-LtxA delivery.

## 2. Results

### 2.1. Purification and Characterization of A. actinomycetemcomitans JP2 Outer Membrane Vesicles (OMVs)

**Figure 1:**
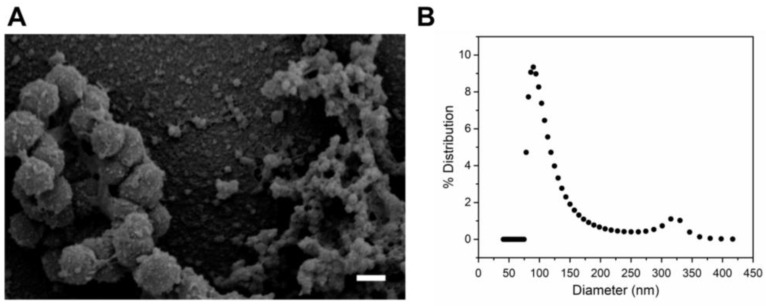
Characterization of *A. actinomycetemcomitans* JP2 OMVs. (**A**) Scanning electron microscope (SEM) image of *A. actinomycetemcomitans* JP2 outer membrane vesicles (OMVs). A heterogeneous population was observed, with one population in the 300 nm diameter range and the second population in the 60–100 nm diameter range. Scale bar: 300 nm. (**B**) Dynamic light scattering (DLS) distribution of OMV sizes obtained from a regularized fit. Two populations of OMV sizes were detected, an abundant population of small (100 nm) OMVs and a less abundant population of large (325 nm) OMVs.

*A. actinomycetemcomitans* was grown to the late log phase, and OMVs were isolated from the cell-free supernatant. Scanning electron microscopy (SEM) analysis of these OMVs showed a heterogeneous population of spherical vesicles, one population with diameters of approximately 300 nm and another population with diameters of approximately 60 nm (Figure 1A). Dynamic light scattering (DLS) was used to substantiate the diameters visualized by SEM. The number-weighted probability density in Figure 1B likewise shows a heterogeneous distribution, with one abundant, small (100 nm) population and a second less abundant, large (325 nm) population, consistent with the SEM results.

### 2.2. Association of LtxA with JP2 OMVs

**Figure 2:**
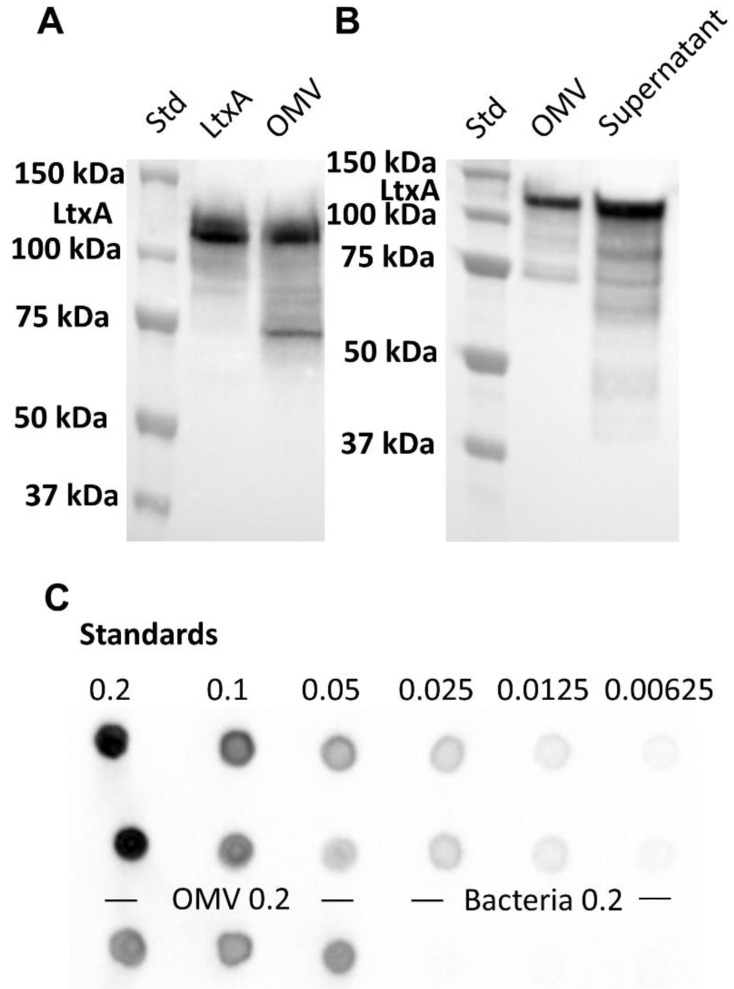
Association of LtxA with OMVs. (**A**) Western blot to detect LtxA on purified OMVs (lane 3) confirms that the vesicles contain full length LtxA (114 kDa). For comparison, purified LtxA is included in lane 2. (**B**) Western blot for LtxA comparing purified OMVs with the remaining supernatant (after ultracentrifugation). OMVs and OMV-free supernatant were diluted to the same volume to allow a direct comparison of the LtxA content in both fractions. Intact LtxA was detected in both the OMVs and OMV-free supernatant, with OMVs containing approximately one-third of the total secreted LtxA. (**C**) Dot blot comparisons of the LtxA concentration in OMVs and bacterial cells. The OMVs and bacteria were lysed then spotted on a nitrocellulose membrane at A_280_ values of 0.2, as marked. Standards of purified LtxA were also spotted at A_280_ values of 0.2, 0.1, 0.05, 0.025, 0.0125, and 0.00625. ImageJ was used to develop a standard curve of spot intensity versus concentration, which was then used to calculate the LtxA composition of the OMVs and cells.

To determine if JP2 OMVs contain full-length LtxA, a Western blot was conducted. As shown in Figure 2A, a strong antibody-reactive band with a molecular weight of 114 kDa was detected in the OMV fraction (lane 3), identical to the purified LtxA band (lane 2), demonstrating that full-length LtxA is associated with the vesicles. An additional, smaller band was detected in the OMV fraction, which likely represents a fragment of LtxA containing the C-terminal portion of the protein (as the antibody used recognizes an epitope in this region of the protein.) A second Western blot was conducted to compare the relative amounts of LtxA present in the JP2 supernatant in its free and OMV-associated forms. OMVs were purified by ultracentrifugation, and the pellet containing OMVs was resuspended in 4 mL phosphate buffered saline (PBS). The OMV-free supernatant (4 mL) was also collected. Both samples were then analyzed by Western blotting using an anti-LtxA antibody [57]. As shown in Figure 2B, the OMV fraction contained a significant amount of full-length LtxA. Densitometry analysis using ImageJ was used to determine that the OMVs contained approximately one-third of the total secreted LtxA. 

We next conducted a dot blot to compare the composition of LtxA in both the OMVs and *A. actinomycetemcomitans* cells (mass LtxA/mass total protein). Purified LtxA at known protein concentrations, determined by measuring the absorbance at a wavelength of 280 nm (A_280_), was spotted onto a nitrocellulose membrane. JP2 OMVs, purified by ultracentrifugation, and cells were diluted to the same protein concentrations, corresponding to an A_280_ of 0.2, lysed, and then spotted on the same membrane. Detection of LtxA was accomplished using a monoclonal anti-LtxA antibody [57] followed by GAM-HRP (Figure 2C). ImageJ software was used to measure the intensity of the LtxA spots to create an intensity vs. LtxA concentration curve to which the intensity of the OMV and cell spots could be compared. Using this method, we calculated that LtxA comprised approximately 40% (by mass) of the protein composition of the JP2 OMVs but only 2% of the protein composition of the JP2 cells.

**Figure 3:**
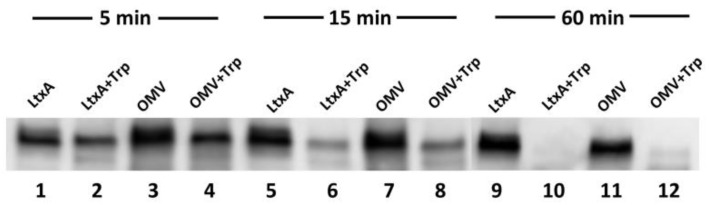
Western blot for LtxA following trypsin digestion of free and OMV-associated LtxA. Purified LtxA was untreated (lanes 1, 5, and 9) or incubated with trypsin for 5, 15, or 60 min (lanes 2, 6, and 10, respectively). Similarly, OMVs were untreated (lanes 3, 7, and 11) or incubated with trypsin for 5, 15, or 60 min (lanes 4, 8, and 12, respectively). No protection of OMV-associated LtxA from trypsin digestion was observed.

LtxA has been reported to bind to the *A. actinomycetemcomitans* cell surface through interactions with nucleic acids present on the surface [58]. Therefore, we hypothesized that LtxA might reside on the surface of the OMVs. To determine if this is the case, or if LtxA is located within the lumen of the OMVs, a trypsin digestion experiment was performed. Both free LtxA and purified OMVs were incubated with trypsin to digest any exposed LtxA. Figure 3 shows the Western blot of untreated LtxA, trypsin-treated LtxA, untreated OMVs, and trypsin-treated OMVs. The trypsin digestion was performed for 5, 15, or 60 min. After a 5-min digestion, 20% of both the free and OMV-associated LtxA was degraded (lanes 1–4). After a 15-min digestion, approximately 60% of both forms of LtxA was degraded (lanes 5–8). After 60 min, most of the toxin was digested (lanes 9–12). This result demonstrates that in the OMV, LtxA resides in a location where it is accessible to trypsin, likely the surface of the vesicle.

### 2.3. OMVs Associate with Host Cells

**Figure 4:**
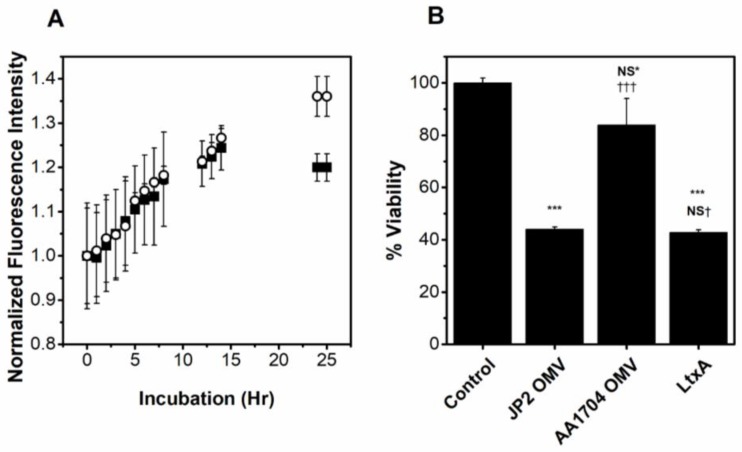
Role of LtxA in OMV association and toxicity. (**A**) Association of JP2 (**⬛**) and AA1704 (⚪) OMVs with THP-1 cells. ANOVA indicates no significant difference in the two data sets. (**B**) Activity of OMV-associated LtxA. THP-1 cells were incubated with PBS, JP2 OMVs, AA1704 OMVs, or LtxA for 16 h and viability was measured using an MTT assay. Data represent mean ± standard deviation (SD) with *n* = 5. NS^*^, not significant; ***, *p* < 0.001 relative to phosphate buffered saline (PBS) control. NS^†^, not significant; †††, *p* < 0.001 relative to JP2 OMVs.

To verify that our purified OMVs associate with and are internalized by THP-1 cells, we labeled the OMVs with a self-quenching concentration of R18; association of the OMV with either the plasma membrane or intracellular membranes results in probe dilution and fluorescence dequenching, observed as an increase in fluorescence associated with the cells. To demonstrate that the observed increase in fluorescence is due to OMV association with cells, we have visualized the association using confocal microscopy. R18-OMVs were incubated with THP-1 cells for 0, 1, 2, and 5 h and imaged by confocal microscopy. As shown in Figure S1, little fluorescence is observed at early time-points, but after a 2 h incubation, the R18 dye can be seen on the cell membrane (Figure S1). After a 5 h incubation, the fluorescence is greater and localized throughout the cell. To determine if LtxA is required for OMV association to host cells, we compared the association of purified OMVs produced by an *ltxA* mutant strain AA1704 with the association of purified JP2 OMVs with THP-1 cells. Strain AA1704 is an isogenic mutant of JP2 in which the *ltxA* gene has been knocked out [59]. A slight decrease in the extent of association was observed in the LtxA-free OMVs at later time points, which may be due to the toxicity of the LtxA-containing OMVs at these time points. Additionally, analysis of variance (ANOVA) demonstrated no significant difference in the association of the two types of OMVs. These results are thus consistent with a previous report [60], and indicate that the presence of LtxA does not increase the association of OMVs with host cells (Figure 4A).

### 2.4. OMV-Associated LtxA Is Active

To determine if LtxA associated with JP2 OMVs is active, THP-1 cells were treated with purified OMVs produced by either JP2 or an *ltxA* mutant strain AA1704 [59], at the same concentration as determined using the FM 4-64 lipid dye, for 16 h. As a positive control, purified LtxA at the same concentration as in the JP2 OMVs was also incubated with the cells for 16 h. As shown in Figure 4B, the JP2 OMVs were as toxic to the THP-1 cells as free LtxA, while the AA1704 OMVs, which do not contain LtxA, were only slightly toxic to the host cells. This finding indicates that even in an OMV-associated form, LtxA is active against host cells. LtxA-containing OMVs (JP2) are more toxic than those without LtxA (AA1704), demonstrating that it is the presence of LtxA in the OMVs that mediates this cytotoxicity. 

### 2.5. Inhibitors of LtxA Are Unable to Inhibit OMV-Associated LtxA

We have previously developed inhibitors of LtxA, based on our knowledge of free LtxA trafficking to host cells [54,55,56]. Because a large fraction of secreted LtxA is associated with OMVs, we investigated whether these inhibitors might be effective in preventing (1) association of the OMVs with host cells and (2) OMV-LtxA-mediated cytotoxicity.

**Figure 5:**
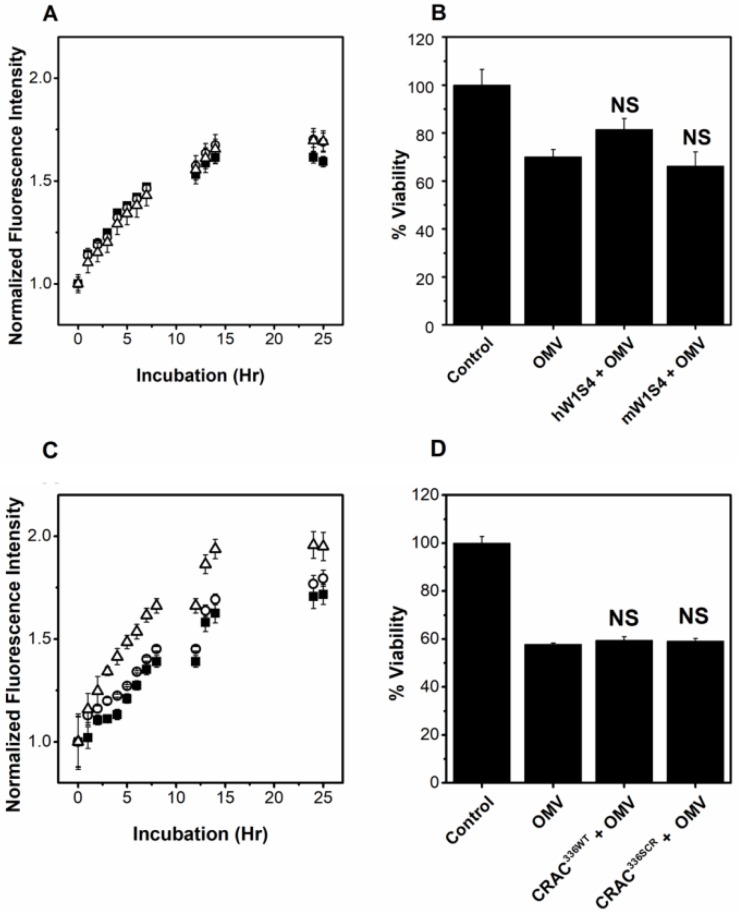
Inhibition of OMV-LtxA by anti-LtxA peptides. (**A**) Association with THP-1 cells by OMVs pretreated with no peptide (**⬛**), hW1S4 peptide (human CD11a analog) (⚪), or mW1S4 (mouse CD11a analog) (△). No significant difference in the association of OMVs in the presence or absence of hW1S4 and mW1S4 were observed as determined by ANOVA. (**B**) Viability of THP-1 cells after incubation with untreated OMVs or OMVs pretreated with the hW1S4 or mW1S4 peptides. (**C**) Association of OMVs with THP-1 cells that were untreated (**⬛**), pretreated with CRAC^336WT^ peptide (⚪), or pretreated with CRAC^336SCR^ peptide (△). No significant difference between the CRAC^336WT^ and control data was determined by ANOVA. The CRAC^336SCR^ data is significantly different (***, *p* < 0.001) than both the control and CRAC^336WT^ data, as determined by ANOVA. (**D**) OMV-mediated cytotoxicity against THP-1 cells that were untreated or pretreated with the CRAC^336WT^ or CRAC^336SCR^ peptides. NS, not significant relative to OMV-treated cells.

To inhibit binding of LtxA to LFA-1, our lab has developed a peptide, hW1S4, which is derived from one of the reported LtxA binding sites of LFA-1 [14] and specifically inhibits LtxA association to the β-propeller domain of the CD11a subunit of LFA-1 [56]. Additionally, we have created a control peptide, mW1S4, based on the analogous murine domain of LFA-1, to which LtxA is unable to bind and which does not inhibit LtxA association with LFA-1 [56]. To determine if inhibition of LtxA binding to LFA-1 prevents OMV association with host cells, R18-labeled OMVs (purified by ultracentrifugation) were preincubated with one of the two peptides for 30 min to block the LFA-1 binding sites of LtxA and then incubated with THP-1 cells at 37 °C. Figure 5A shows an increase in cellular fluorescence through approximately 15 h of incubation, demonstrating association of the OMVs with the cells under all conditions. The association of untreated, hW1S4-treated and mW1S4-treated OMVs with THP-1 cells is similar, suggesting that, unlike free LtxA, the process of cellular association is not mediated by recognition of the CD11a subunit of LFA-1 on the host cell by LtxA on the OMV surface.

We also investigated whether the hW1S4 peptide could inhibit OMV-associated LtxA-mediated cytotoxicity. As shown in Figure 5B, pretreatment of the purified OMVs with the hW1S4 peptide slightly decreased OMV-mediated cytotoxicity; however, this inhibition was not statistically significant. The mW1S4 peptide had no effect on OMV-mediated cytotoxicity. Together, these results indicate that our targeted inhibitor of LtxA binding to LFA-1 is ineffective in preventing OMV-associated LtxA from interacting with the host cells.

We next investigated whether our inhibitor of LtxA binding to cholesterol could inhibit toxin delivery when it is in an OMV-associated form. To inhibit free LtxA binding to cholesterol, we have developed a cholesterol-binding CRAC^336WT^ peptide, which contains the cholesterol-binding motif of LtxA and retains the cholesterol-binding activity of the toxin [54,55]. In addition, we have developed a control peptide, CRAC^336SCR^, which contains a scrambled CRAC motif and thus does not bind cholesterol or inhibit LtxA activity. In these experiments, THP-1 cells were incubated with one of the peptides or PBS for 30 min before incubation with R18-labeled OMVs. As shown in Figure 5C, the fluorescence of cells treated with R18-OMVs increased with time, demonstrating OMV association, with a plateau in fluorescence at a time of approximately 20 h. The CRAC^336WT^ peptide demonstrated no inhibitory effect on the OMV association with THP-1 cells, while the CRAC^336SCR^ peptide slightly increased OMV association with THP-1 cells.

We then investigated whether the CRAC^336WT^ peptide had an effect on OMV-mediated cytotoxicity. As in the association experiments, the THP-1 cells were treated with either CRAC^336WT^ or CRAC^336SCR^, or untreated, and then incubated with purified OMVs. The viability of the OMV-treated cells was calculated after 16 h of incubation with OMVs. As shown in Figure 5D, the OMV-mediated cytotoxicity was approximately 50%. Neither the CRAC^336WT^ nor CRAC^336SCR^ peptide inhibited this toxicity.

### 2.6. OMV-Associated LtxA Is Trafficked to Host Cells in an LFA-1- and Cholesterol-Independent Mechanism

Our results with our targeted inhibitors suggest that in its OMV-associated form, LtxA does not recognize the same molecules on the host cell surface as it does in its free form. To verify this, we conducted a series of experiments to determine whether LFA-1 and cholesterol are involved in the OMV-LtxA trafficking process.

**Figure 6:**
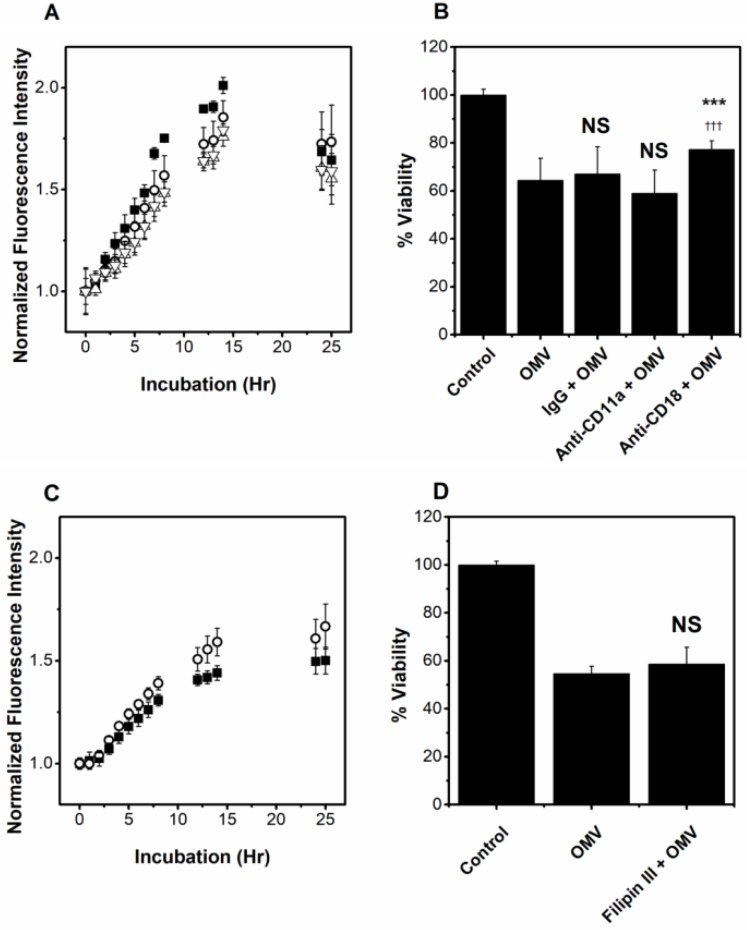
Role of LFA-1 and Cholesterol in OMV delivery and activity. (**A**) Association of OMVs with THP-1 cells that were untreated (**⬛**), or pretreated with IgG (⚪), anti-CD11a (△), or anti-CD18 (▽) monoclonal antibodies. Data represent mean ± SD with *n* = 5. No significant difference in the association of the OMVs in the presence of either anti-LFA-1 antibody was observed relative to the IgG control, as determined using ANOVA. (**B**) Viability of THP-1 cells after pretreatment with IgG, anti-CD11a, or anti-CD18 monoclonal antibodies. Data represent mean ± SD with *n* = 6. NS, not significant (*p* > 0.01) relative to OMV control; ***, *p* < 0.001 relative to OMV treatment; †††, *p* < 0.001 relative to IgG treatment. (**C**) Association of OMVs with THP-1 cells that were untreated (**⬛**) or pretreated with filipin III (⚪). Data represent mean ± SD with *n* = 5. No significant difference in association in the presence or absence of filipin III was observed as determined by ANOVA. (**D**) OMV-mediated cytotoxicity against THP-1 cells that were untreated or pretreated with filipin III. Data represent mean ± SD with *n* = 6. NS, not significant (*p* > 0.01) relative to OMV control.

It has been reported that LtxA recognizes the integrin-epidermal growth factor-like (I-EGF) domains of the CD18 subunit of LFA-1 [61]. To investigate the role of LtxA recognition of this subunit in OMV association with host cells, we blocked this binding domain using an anti-CD18 (MEM-48) antibody, which recognizes an epitope located between residues 534–546 of the CD18 subunit [62] and has previously been shown to inhibit LtxA-mediated cytotoxicity in HL-60 cells [63]. Additionally, we used a monoclonal antibody against CD11a (EP1285Y), which recognizes a domain of LFA-1 that is C-terminal to the β-propeller domain to investigate the role of other CD11a domains in OMV association. IgG was used as a control. We incubated THP-1 cells for 30 min with the antibody before adding the R18-labeled OMVs. As shown in Figure 6A, the effect of both anti-CD11a and anti-CD18 antibodies on OMV association with THP-1 cells was similar to that of the IgG control, demonstrating that blocking either subunit of LFA-1 has no effect on the ability of the OMVs to bind to the cell. The anti-CD11a antibody did not inhibit OMV-mediated cytotoxicity, while the anti-CD18 antibody exhibited a slight but statistically significant inhibition of OMV-mediated cytotoxicity (Figure 6B). In contrast to this slight inhibition of activity, this antibody has been shown to completely abolish the cytotoxic activity of soluble LtxA [63].

The role of cholesterol in OMV association with host cells was assessed by investigating R18-OMV association over time, after inhibiting cholesterol-rich lipid raft formation in THP-1 cells by filipin III [64]. THP-1 cells were pretreated with filipin III for 30 min and then with R18-OMVs. Filipin III increased, rather than decreased, the association of JP2 OMVs with THP-1 cells (Figure 6C), but had no significant effect on OMV-mediated cytotoxicity (Figure 6D).

### 2.7. OMV-Associated LtxA Is Active against Cells That Lack LFA-1

**Figure 7:**
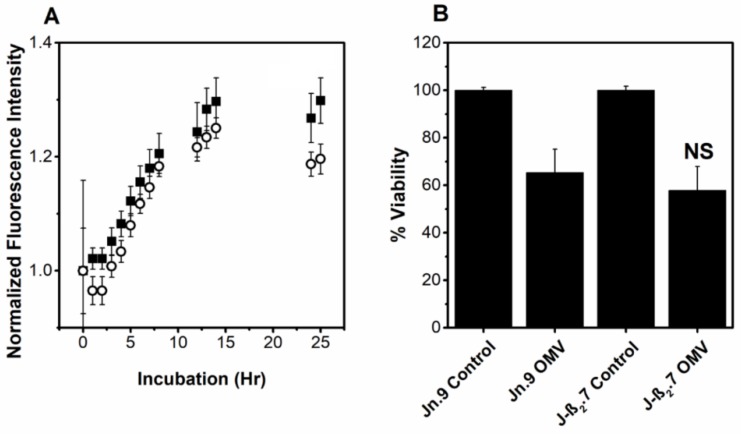
Interaction of JP2 OMVs with non-LFA-1-expressing cells. (**A**) Association of OMVs with Jn.9 cells, which express LFA-1 (⬛) and J-β_2_.7 cells, which do not express the CD11a subunit of LFA-1, resulting in non-functional LFA-1 (⚪). No significant difference in the association was observed as determined by ANOVA. (**B**) OMV-mediated cytotoxicity against Jn.9 and J-β_2_.7 cells. Data represent mean ± SD with *n* = 6. NS, not significant relative to Jn.9 OMVs.

Together, our results demonstrate that inhibition of OMV-associated LtxA binding to LFA-1 and cholesterol has little effect on the association of the OMVs with the cells and the resulting cytotoxicity. The independence of this mechanism on LFA-1 suggests that OMV-associated LtxA may be active in cells which are not susceptible to free LtxA. To investigate this, we compared the response to OMVs of two Jurkat cell lines: Jn.9, which express CD11a, and J-β_2_.7, which do not express CD11a [65]. The association of the OMVs to the Jn.9 cells expressing intact LFA-1 was greater than the association of the OMVs with J-β_2_.7 cells, which lack functional LFA-1 (Figure 7A). However, the difference was small, and the OMV-mediated cytotoxicity in both cell lines was not significantly different (Figure 7B). In contrast to these results, soluble LtxA is entirely ineffective in killing the J-β_2_.7 cells [14]. This result indicates that OMV-associated LtxA is active against a wider range of cells than free LtxA, as its activity is LFA-1-independent.

## 3. Discussion

In this study, we demonstrated that a large fraction of the LtxA present in the JP2 bacterial supernatant is associated with OMVs. Our results also demonstrated that the OMV-associated LtxA is located entirely on the surface of the OMV, which is consistent with previous reports of the toxin’s strong affinity for the bacterial cell surface [58,66,67]. Importantly, we found that in this OMV-associated form, although LtxA is on the surface of the vesicle, the OMV is delivered to THP-1 cells in a cholesterol- and LFA-1-independent mechanism, in contrast to delivery of free LtxA, which requires both cholesterol and LFA-1 for host cell intoxication [14,15,16]. We propose that the LFA-1-independence of this interaction may allow the toxin to be trafficked to host cells beyond just those that express LFA-1, possibly expanding the role of LtxA in *A. actinomycetemcomitans* virulence.

**Figure 8:**
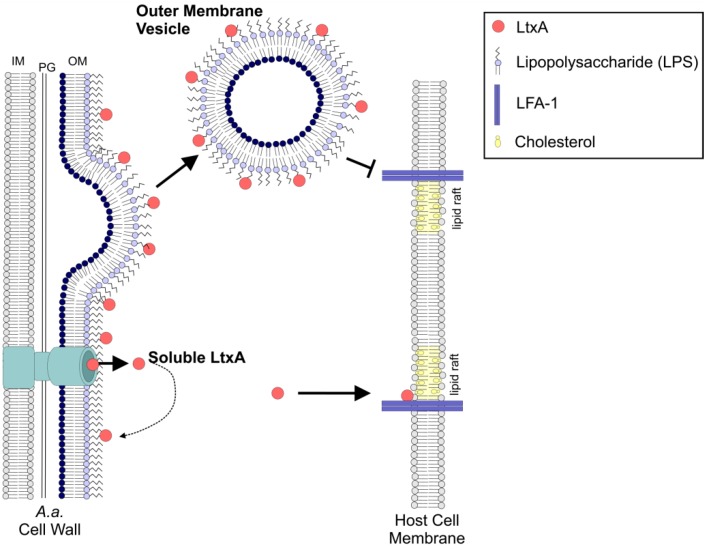
A model of LtxA association with OMVs and the lipid raft- and LFA-1-independent delivery to host cells. In this model, LtxA is secreted by *A. actinomycetemcomitans* through a one-step Type 1 secretion system across the inner and outer membranes. After being released into the extracellular environment, a significant fraction of the LtxA reassociates with the bacterial surface due to electrostatic interactions. As OMVs form from the outer membrane, LtxA is incorporated on the surface of the vesicle.

A number of RTX toxins have been reported to be delivered to host cells via OMVs, including *Bordetella pertussis* adenylate cyclase toxin (ACT), *Kingella kingae* RtxA, and enterohemorraghic *Escherichia coli* (EHEC) hemolysin (Hly) [28,29,68]. Like OMV-LtxA, OMV-ACT was found to be trypsin sensitive [29], indicating that it is located on the surface of the vesicles. Similarly, EHEC-Hly was found in low concentrations in the OMV-free supernatant due to rapid binding to the exterior of OMVs [25,68]. Our results, in combination with these previously reported results, suggest a common trend for the fate of RTX toxins secreted by a Type I secretion system in which, after secretion across the inner and outer membranes via Type I secretion, some fraction of the toxin associates with the bacterial cell or OMV surface rather than being released into the environment in its free form. As a result, these toxins are found on the surface of the OMVs. Our working model of LtxA delivery to THP-1 cells by OMVs is shown in Figure 8.

Significant variation in the reported mechanisms by which OMVs are internalized by host cells exists in the literature. Lipid raft- and receptor-mediated processes have been reported [69,70] as well as fusion [71]. In many cases, the delivery of an OMV-associated toxin differs from that of the free toxin. For example, OMV-associated ACT is reported to require cholesterol [29] but not the CD11b integrin that free ACT requires [72]. Our results have demonstrated that although free LtxA has an absolute requirement for both cholesterol and LFA-1 on the host cell [14,15,16,19], the activity of OMV-associated LtxA is mostly independent of these two factors.

Recently, it has been proposed that this variation in reported mechanisms of OMV internalization may be due to variations in OMV sizes in addition to species-dependent differences in protein composition [43]. Interestingly, we found that OMVs purified from the late log phase of *A. actinomycetemcomitans* strain JP2 were heterogeneous in diameter, with one population of approximately 100 nm and another of approximately 300 nm. Both populations are within the previously reported size range of OMVs produced by *A. actinomycetemcomitans* and other species. For example, *A. actinomycetemcomitans* strain D7SS OMVs are heterogeneous in size, with sizes similar to what we observed in JP2 [48]. Our future work aims to refine the current understanding of *A. actinomycetemcomitans* OMV delivery by investigating the size-dependence of this process.

We suspect that one of the common purposes of OMVs is to provide an alternative pathway for the delivery of toxin, perhaps to broaden the range of activity, as we have proposed here for LtxA. We have previously reported that *Vibrio cholerae* OMVs, which contain cholera toxin (CT), are internalized in a GM1-independent manner [73], even though soluble CT requires this ganglioside receptor. OMVs produced by *A. actinomycetemcomitans* strain D7SS (serotype a) were observed to contain only the A and B subunits of Cdt but retain the cytolethal distending activity of Cdt [48]; because both A and C subunits are required for the cholesterol-dependent binding of the holotoxin [74,75,76,77], this finding suggests that OMV-mediated delivery of this toxin likely occurs through entirely different mechanisms than that of the purified, soluble toxin, as well.

In conclusion, this work demonstrates that LtxA is secreted in two distinct forms, a free form that requires LFA-1 and cholesterol and an OMV-associated form that does not. We propose that OMV-association provides the toxin with an ability to interact with multiple host cell types, thus increasing the activity of this toxin. Our current work is focused on identifying the molecules and pathways involved in OMV-LtxA delivery so that we can design inhibitors of this form of toxin to develop new therapeutic strategies for LAP.

## 4. Materials and Methods

### 4.1. Bacterial Strain Cultivation

*A. actinomycetemcomitans* strains JP2 (serotype b) [68] and an its *ltxA* mutant, AA1704 [59], were grown in a candle jar in 30 g/L trypticase soy broth (BD Biosciences, Franklin Lakes, (NJ,) USA) with 6 g/L yeast extract (BD Biosciences), supplemented with 0.4% sodium bicarbonate (Fisher Scientific, Hampton, (NH,) USA), 0.8% dextrose (BD Biosciences), 5 µg/mL vancomycin (Sigma-Aldrich, St. Louis, (MO,) USA), and 75 µg/mL bacitracin (Sigma-Aldrich).

### 4.2. Cell Culture

THP-1 leukocytes (ATCC, Manassas, (VA,) USA) were maintained at 37 °C in 5% CO_2_ in RPMI 1640 medium (ThermoFisher Scientific, Waltham, (MA,) USA), supplemented with 10% fetal bovine serum (FBS, Quality Biological, Gaithersburg, (MD,) USA) and 0.05 mM 2-mercaptoethanol (VWR, Radnor, (PA,) USA). Two Jurkat cell lines, Jn.9 and the CD11a-deficient mutant, J-β_2_.7 [66] (gifts from Dr. Edward Lally, University of Pennsylvania), were cultured in RPMI 1640 medium supplemented with 10% FBS, 0.1 mM MEM non-essential amino acids, 1x MEM vitamin solution, 2 mM L-glutamine, and 50 µg/mL gentamicin [18].

### 4.3. Purification of OMVs

All OMVs used in this study were purified as described previously [78]. The bacteria were grown to the late exponential phase, then centrifuged twice at 10,000× *g* for 10 min. The supernatant was then filtered through at 0.45 µm filter to remove any remaining bacteria. The bacteria-free supernatant was ultracentrifuged at 105,000× *g* for 30 min, and the pellets were pooled in phosphate-buffered saline (PBS, pH 7.4) before the ultracentrifuge step was repeated.

The presence of LtxA in the purified OMVs was verified using sodium dodecyl sulfate-polyacrylamide gel electrophoresis (SDS-PAGE) followed by Western blotting using anti-LtxA antibody [57]. First, the proteins in the OMV sample were separated on an 8.5% acrylamide mini-protean TGX precast gels (Bio-Rad, Hercules, (CA,) USA), then transferred to a nitrocellulose membrane (Bio-Rad). LtxA was detected using a monoclonal anti-LtxA antibody [57], followed by a horseradish peroxidase-conjugated goat anti-mouse (GAM-HRP) secondary antibody. Detection was completed using SuperSignal™ West Dura substrate (ThermoFisher Scientific).

To track OMV association with host cells, purified OMVs were labeled with a self-quenching concentration (0.5 mg/mL) of octadecyl rhodamine B chloride (R18, ThermoFisher Scientific) [71]. To compare the lipid content between OMV strains, FM 4-64™ dye (ThermoFisher Scientific) was used. The fluorescence of the labeled OMVs, which is a measure of lipid content, was measured at an excitation wavelength of 515 nm and emission wavelength of 640 nm using a Tecan plate reader.

### 4.4. LtxA Purification

LtxA was purified from the *A. actinomycetemcomitans* culture supernatant, as described previously [63]. The purity of LtxA was confirmed by SDS-PAGE and the identity was confirmed by Western blot, using an anti-LtxA monoclonal antibody [57]. Activity was confirmed using a cytotoxicity assay.

### 4.5. Peptide Synthesis

Peptides were synthesized as described previously [54,55,56] using 9-fluorenylmethylcarbonyl (Fmoc) solid-phase synthesis.

### 4.6. Dynamic Light Scattering (DLS)

An ALV/CGS-3 goniometer system was used to determine the average size of the vesicles. OMV samples suspended in PBS were measured three times for two minutes each at a wavelength of 632.8 nm and at a 90° scattering angle. Vesicle size distributions were calculated using a number-weighted regularized fit with the coated sphere assumption [79] and a membrane thickness (r*) of 5 nm [80] using the ALV software ( ALV-5000/E, ALV-GmbH, Langen, Germany, 2001)

### 4.7. Scanning Electron Microscopy (SEM)

Glass slides (Electron Microscopy Sciences, Hatfield, (PA,) USA) were plasma-cleaned, and the vesicles were dried and fixed with Karnovsky’s fixative [81]. The vesicles were sequentially dehydrated in 35, 70, 85, 95, and 100% ethanol solutions followed by 50:50 hexamethyldisilazane (HMDS):ethanol (Sigma-Aldrich) and finally 100% HMDS [82]. The samples were coated with iridium and visualized on a Hitachi 4300 scanning electron microscope at an accelerating voltage of 3 kV.

### 4.8. Immunoblotting

To determine the LtxA composition of *A. actinomycetemcomitans* OMVs and cells, a dot blot was performed by first lysing samples with 0.5% Triton-X 100 (Sigma-Aldrich). Next, 2 µL volumes of the samples, each containing 0.2 mg/mL total protein, determined by the absorbance at a wavelength of 280 nm (A_280_), were blotted onto a nitrocellulose membrane (Bio-Rad). Known concentrations of purified LtxA were also blotted onto the membrane as standards. The membrane was dried and blocked in blotto solution (5% dried milk in Tris-buffered saline with 0.1% Tween-20 (TBST)) for 1 h. The presence of LtxA was detected with a monoclonal anti-LtxA antibody [57], followed by goat anti-mouse horseradish peroxidase (GAM-HRP).

### 4.9. Trypsin Digestion

A trypsin protection assay was performed to determine the location of LtxA within the OMVs. LtxA and OMVs were treated with 0.04 mg/mL trypsin (Fisher Scientific) for 5, 15, or 60 min at 37 °C in PBS, pH 7.4. The trypsin was inactivated using 2% formic acid. The proteins were separated using SDS-PAGE on a 4–15% acrylamide Mini-PROTEAN^®^ TGX™ precast gel (Bio-Rad). Following transfer to a nitrocellulose membrane, LtxA was detected using an anti-LtxA antibody [57] followed by GAM-HRP.

### 4.10. Confocal Imaging

Confocal images were collected using a Nikon Eclipse Ti microscope at 60× magnification. THP-1 cells were added to poly-L-lysine (PLL)-coated plates at 40,000 cells/mL and allowed to settle for 30 min. The nucleus of the cells was stained using NucBlue™ Live ReadyProbes™ Reagent (ThermoFisher Scientific, blue). R18-OMVs (red) were incubated with the cells at a volume corresponding to an approximate concentration of 1 µg LtxA and tracked as they interacted with the cells over 5 h using a Nikon C2si+ confocal microscope with a 60× oil immersion objective using 405 nm and 561 nm laser sources.

### 4.11. OMV Association Assay

To follow the kinetics of OMV association with THP-1 cells, R18-OMVs were incubated with THP-1 cells at a volume corresponding to an approximate concentration of 1 µg LtxA per 1 million THP-1 cells or 2 µg LtxA per one million Jn.9/J-β_2_.7 cells. (The OMV concentrations varied between the different cell lines because of varying susceptibility to LtxA; the concentrations were adjusted to result in a similar amount of cell death after 16 h of exposure to the OMVs.) All incubations were conducted at 37 °C under 5% CO_2_. The increase in fluorescence due to dye dilution as the OMVs associated with the cells was tracked over 25 h using a plate reader (Tecan, Männedorf, Switzerland), with an excitation wavelength of 555 nm and an emission wavelength of 588 nm. The fluorescence was normalized to the fluorescence of R18-labeled OMVs incubated with cell-free culture media.

### 4.12. Cytotoxicity Assays

To measure the OMV-mediated cytotoxicity, JP2 OMVs were added to cells at a concentration corresponding to an approximate LtxA concentration of 1 µg LtxA per one million THP-1 cells or 2 µg LtxA per one million Jn.9/J-β_2_.7 cells for 16 h. OMVs from strain AA1704 were incubated with THP-1 cells at a lipid content equal to that of JP2 OMVs, as measured with FM 4-64™ dye. The cellular metabolic activity was then measured using a 3-(4,5-dimethylthiazol-2-yl)-2,5-diphenyltetrazolium bromide (MTT)-based assay. To each sample well, 0.48 mg/mL thiazolyl blue tetrazolium bromide (Sigma-Aldrich) in PBS was added and incubated for 4 h at 37 °C. The media was removed, and the precipitate was dissolved in dimethyl sulfoxide (DMSO); the absorbance was then measured at a wavelength of 570 nm [83]. The absorbance from treated cells compared to the absorbance from non-treated cells was used as a measure of cell viability.

### 4.13. Inhibition of Cholesterol Binding

To inhibit binding of OMVs to plasma membrane cholesterol, THP-1 cells were incubated with either the CRAC^336WT^ peptide, which inhibits binding of LtxA to cholesterol or the CRAC^336SCR^ peptide, a control peptide which does not inhibit binding of LtxA to cholesterol [55], at a final concentration of 12 µM. After this 30-min incubation, OMVs were added at a concentration corresponding to an LtxA concentration of 1 µg LtxA per 1 million THP-1 cells. These concentrations and toxin:peptide ratios were equal to those used previously in our experiments with free LtxA [55]. In a subsequent set of experiments, THP-1 cells were pre-incubated for 30 min with 10 µg/mL filipin III (Sigma-Aldrich) [48] to disrupt cholesterol-rich lipid rafts before incubation with OMVs at the same concentration.

### 4.14. Inhibition of LFA-1 Binding

To inhibit binding of OMVs to LFA-1, OMVs were preincubated for 30 min with either the hW1S4 peptide, analogous to a portion of the β-propeller of the CD11a subunit of human LFA-1, which inhibits LtxA binding to LFA-1, or the mW1S4 peptide, corresponding to the same domain of murine LFA-1 and which does not inhibit LtxA binding to LFA-1 [56]. Both peptides were used at a concentration of 1.73 µM. The peptide-treated OMVs were then added to the cells at a concentration corresponding to 1 µg LtxA per 1 million THP-1 cells. These concentrations and toxin:peptide ratios were equal to those used in our previous experiments with free LtxA [56]. Additionally, in a separate set of experiments, the CD11a or CD18 subunits of the LFA-1 integrin were blocked with anti-CD11a (EP1285Y, ab52895) or anti-CD18 (MEM-48, ab657) monoclonal antibodies (Abcam, Cambridge, (MA,) USA) or with a nonspecific rabbit immunoglobulin G (IgG) (EPR25A, ab172730) for 30 min at a concentration of 1 ng/mL before incubation with the OMVs.

### 4.15. Statistical Analysis

Data are presented as the mean ± standard deviation, with the noted sample size in the figure legend. The significance of association data sets was determined with a two-way ANOVA using OriginPro. The significance of the toxicity results was determined using a two-tailed Student’s t-test function. P-values less than 0.01 were considered to be statistically significant. Levels of significance are noted in each figure legend.

## Supplementary Materials

The following are available online at http://www.mdpi.com/2072-6651/10/10/414/s1, Figure S1: OMV association with THP-1 cells.
