# Supplementary material for: Aggregatibacter actinomycetemcomitans Leukotoxin Is Delivered to Host Cells in an LFA-1-Indepdendent Manner When Associated with Outer Membrane Vesicles
Source: Toxins (Basel). 2018 Oct 13;10(10):414. doi: 10.3390/toxins10100414 (PMC6215133; doi:10.3390/toxins10100414)
Supplement: Supplementary file 1 [file toxins-10-00414-s001.pdf]

Supplementary Materials: *Aggregatibacter actinomycetemcomitans* Leukotoxin Is Delivered to Host Cells in an LFA-1-Independent Manner When Associated with Outer Membrane Vesicles

Justin B. Nice, Nataliya V. Balashova, Scott C. Kachlany, Evan Koufos, Eric Krueger, Edward T. Lally, Angela C. Brown

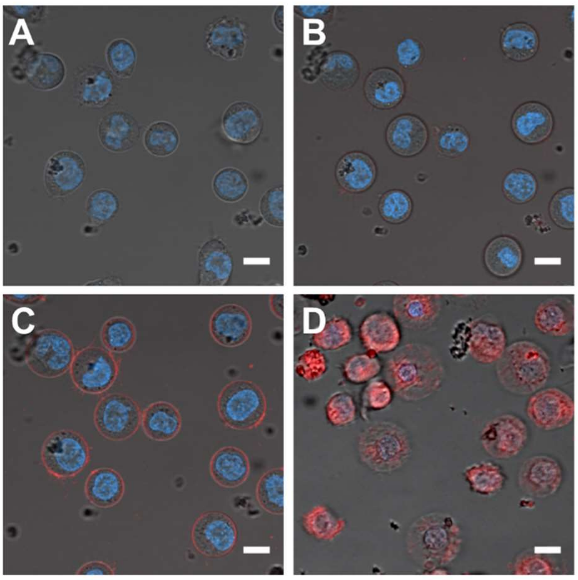

Figure S1. OMV association with THP-1 cells. R18-OMVs were incubated with THP-1 cells for 0 h (**A**), 1 h (**B**), 2 h (**C**), or 5 h (**D**) and imaged by confocal microscopy. At the initial time points (0 h and 1 h), very little cell-associated fluorescence is observed. R18-OMVs can be seen to be associated with the cell membrane at the 2 h time point. After a 5 h incubation, the fluorescence is observed throughout the cells. Scale bar = 100 μm.
